# Using open-source data to construct 20 metre resolution maps of children’s travel time to the nearest health facility

**DOI:** 10.1038/s41597-022-01274-w

**Published:** 2022-05-17

**Authors:** Gary R. Watmough, Magnus Hagdorn, Jodie Brumhead, Sohan Seth, Enrique Delamónica, Charlotte Haddon, William C. Smith

**Affiliations:** 1 Data for Children Collaborative with UNICEF, Edinburgh, United Kingdom; 2 School of GeoSciences, University of Edinburgh, Edinburgh, United Kingdom; 3 Global Academy of Agriculture and Food Systems, University of Edinburgh, Edinburgh, United Kingdom; 4 School of Informatics, University of Edinburgh, Edinburgh, United Kingdom; 5 Moray House School of Education and Sport, University of Edinburgh, Edinburgh, United Kingdom; 6 Division of Data, Analytics, Planning, and Monitoring, UNICEF, New York, United States

**Keywords:** Health services, Sustainability

## Abstract

Physical access to health facilities is an important factor in determining treatment seeking behaviour and has implications for targets within the Sustainable Development Goals, including the right to health. The increased availability of high-resolution land cover and road data from satellite imagery offers opportunities for fine-grained estimations of physical access which can support delivery planning through the provision of more realistic estimates of travel times. The data presented here is of travel time to health facilities in Uganda, Zimbabwe, Tanzania, and Mozambique. Travel times have been calculated for different facility types in each country such as Dispensaries, Health Centres, Clinics and Hospitals. Cost allocation surfaces and travel times are provided for child walking speeds but can be altered easily to account for adult walking speeds and motorised transport. With a focus on Uganda, we describe the data and method and provide the travel maps, software and intermediate datasets for Uganda, Tanzania, Zimbabwe and Mozambique.

## Abstract (summary)

**Table:** 

Measurement(s)	Walking travel time • Walking travel friction
Technology Type(s)	Cost surface of travel speeds andhealth centre locations as destination points • land cover, digital elevation and road data to estimate the effort or friction of travel
Sample Characteristic - Location	Uganda • Mozambique • Tanzania • Zimbabwe

## Background & Summary

Access to services such as schools, health centres, markets and larger regional towns are important for individual’s wellbeing, health and livelihoods in rural areas of developing countries. Increased travel times to key services can result in decreased utilisation^1,2^ and in Uganda 44.0% of women in rural areas reported that distance to health centre contributed to deciding not to access healthcare for themselves^3^. Access to health centres is a key component of national preparedness for infectious disease planning^4^ and a component of several Sustainable Development Goals (SDGs). Therefore, understanding access to key services at sub-national levels is required for improved delivery. Here we present new data of 20-m spatial resolution travel time estimates to health facilities for Uganda, Zimbabwe, Tanzania and Mozambique. We describe here the data characteristics and method for Uganda and provide access to the data for Uganda, Zimbabwe, Tanzania and Mozambique. It is our intention to provide the travel time maps for all sub-Saharan countries periodically through the Edinburgh data share portal https://datashare.ed.ac.uk/handle/10283/3898.

Utilisation of health facilities can be characterised by a complex mixture of travel time, financial costs involved in attending and travelling to a location, education of parents, quality of previous care and other political and societal barriers sometimes preventing access^5,6^. Here we are focused only on travel time as a physical measure of access. Travel time to key services such as schools, clinics and markets is seen as a key component of rural poverty and a major barrier preventing the realization of the right to health among children and, thus, impact child poverty (SDG 1.2.2). However, little empirical evidence currently exists on the levels to which time to travel to health centre contributes to health and poverty outcomes of populations. Therefore, these data provide an opportunity to establish high-resolution estimates of travel time to explore the impact that it has on socioeconomic outcomes such as poverty.

**Fig. 1:**
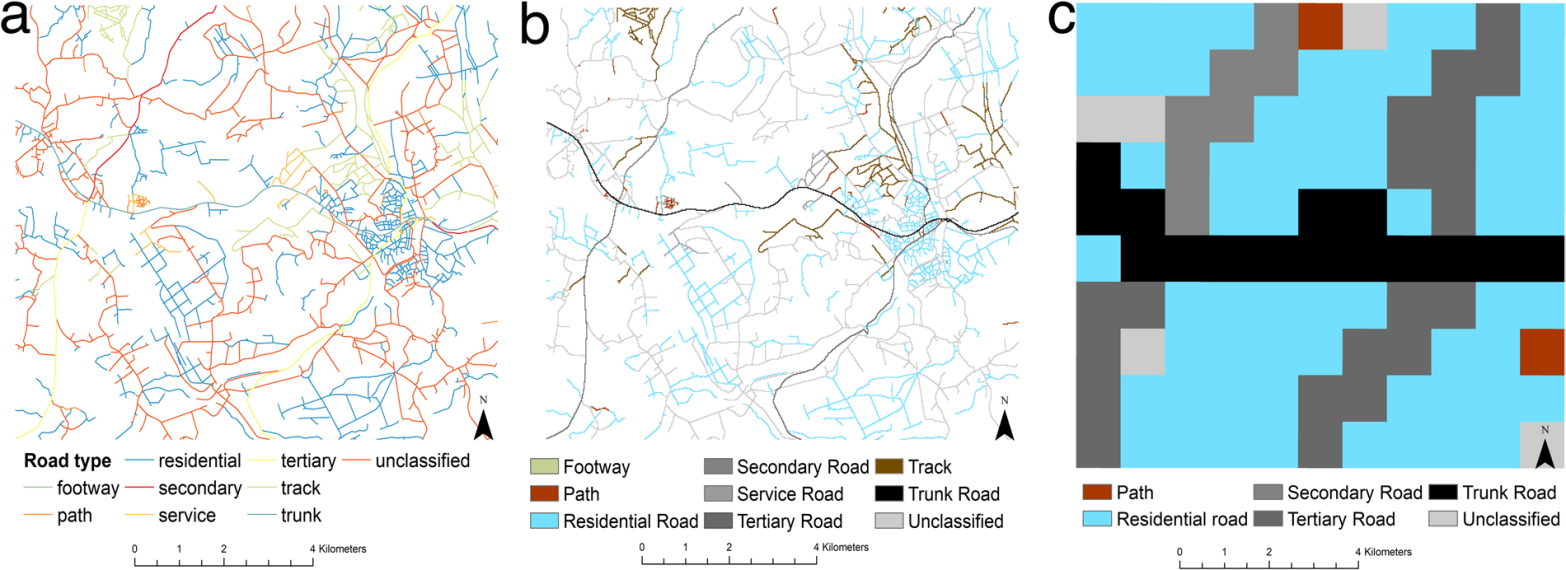
The roads polygon file (**a**) used in the analysis was a combination of Open Street Map and MapwithAi road datasets. The sinuosity of the roads was maintained when using a 20 m raster grid (**b**) compared to a 1 km raster grid (**c**) which is used in other studies.

Measuring distance to health services using straight line or Euclidian distance methods overestimate geographical access or underestimate travel time to services as it does not account for transport routes and barriers to travel such as waterbodies^7–11^. Least cost path estimates are often used to estimate travel time across surfaces^12–14^ as they allow for the mode of transport to be considered^4^. Using a least cost path approach Weiss *et al*. 2020^14^ estimated that approximately 43% of the World’s population could not reach a health facility within 1 hour of walking. Travel time was based on data with a 1 km spatial resolution produced by Weiss *et al*.^15^. The size of cell used in cost surfaces impacts the results with coarser resolution cells leading to the sinuosity of roads being simplified, roads being overrepresented compared with other land cover and the over connection of roads across a landscape (Fig. 1). We developed an estimate of travel time to health centres following published methods^15,16^ using 20 m spatial resolution data. We chose 20 m as it is the finest spatial resolution land cover data available globally at the time of analysis (from Sentinel-2 http://2016africalandcover20m.esrin.esa.int/) and thus is repeatable and transferable. The intention of this study and data is to provide a fine resolution estimation of travel time to service access points to be used by governments and humanitarian agencies such as UNICEF to explore development issues for example, exploring the links between access to health facilities and poverty or planning vaccination rollouts. To the authors’ knowledge we have created the finest spatial resolution travel time data currently available which also uses a more detailed roads dataset from the Mapwith AI project (https://github.com/facebookmicrosites/Open-Mapping-At-Facebook/wiki/Available-Countries) not previously used in travel time map creation.

## Methods

The least cost path method used here can be broken down into two key phases: the first involves creating a ‘cost’ allocation surface which can also be referred to as an effort or friction surface^14^. This represents the effort to travel across a particular pixel and can thus consider the variation in effort (cost) required to travel across different land surfaces. The second phase uses this cost allocation (or effort surface) in a least cost path analysis to estimate travel time from every pixel to the nearest destination location (in this case health centres). This is done using Dijkstra’s algorithm^17^ to create Dijkstra trees which find the shortest path from one point to another^12^. It is implemented here using the graph module of scikit-image in python^18^.

### Creating cost allocation surfaces

The cost allocation or effort surface was assembled using three primary input datasets on land surface characteristics that help or hinder travel speeds: land cover, roads and topography. Initial processing and analysis were undertaken on the individual datasets before being combined into a final cost allocation surface which depicts travel times from health centres across the country. The following sections detail the methodology and technical details of the cost surface creation as well as details on the choices made and research undertaken to feed into the travel time estimates.

### Transport mode choice

There is little published information on how patients in Uganda access health services. However, in Kenya over 80% of patients were found to access health services by walking^19^. To measure travel-time we focused on pedestrian access because non-motorised transport is used for 50% of journeys in Uganda^20^ and at least 50% of households in rural Uganda do not own any form of transport asset^21^. Further, children are most often the responsibility of women but most means of transport in Uganda are owned by men^21,22^. Some women do utilise bicycles, but issues of cost and gender relations make it easier for men to own and use bicycles and there are also negative cultural traditions which inhibit women’s use of transport^20^.

Public transport mostly exists in Uganda through boda bodas (motorcycle taxis) or matutus (shared minibuses). It is likely that when accessing health services further away or when in need of particular medical attention that motorised transport of some form would be used. However, we did not at the time of development have access to transport speed information. Thus, we only consider walking/pedestrian travel in this study.

### Child walking speed definitions

**Fig. 2:**
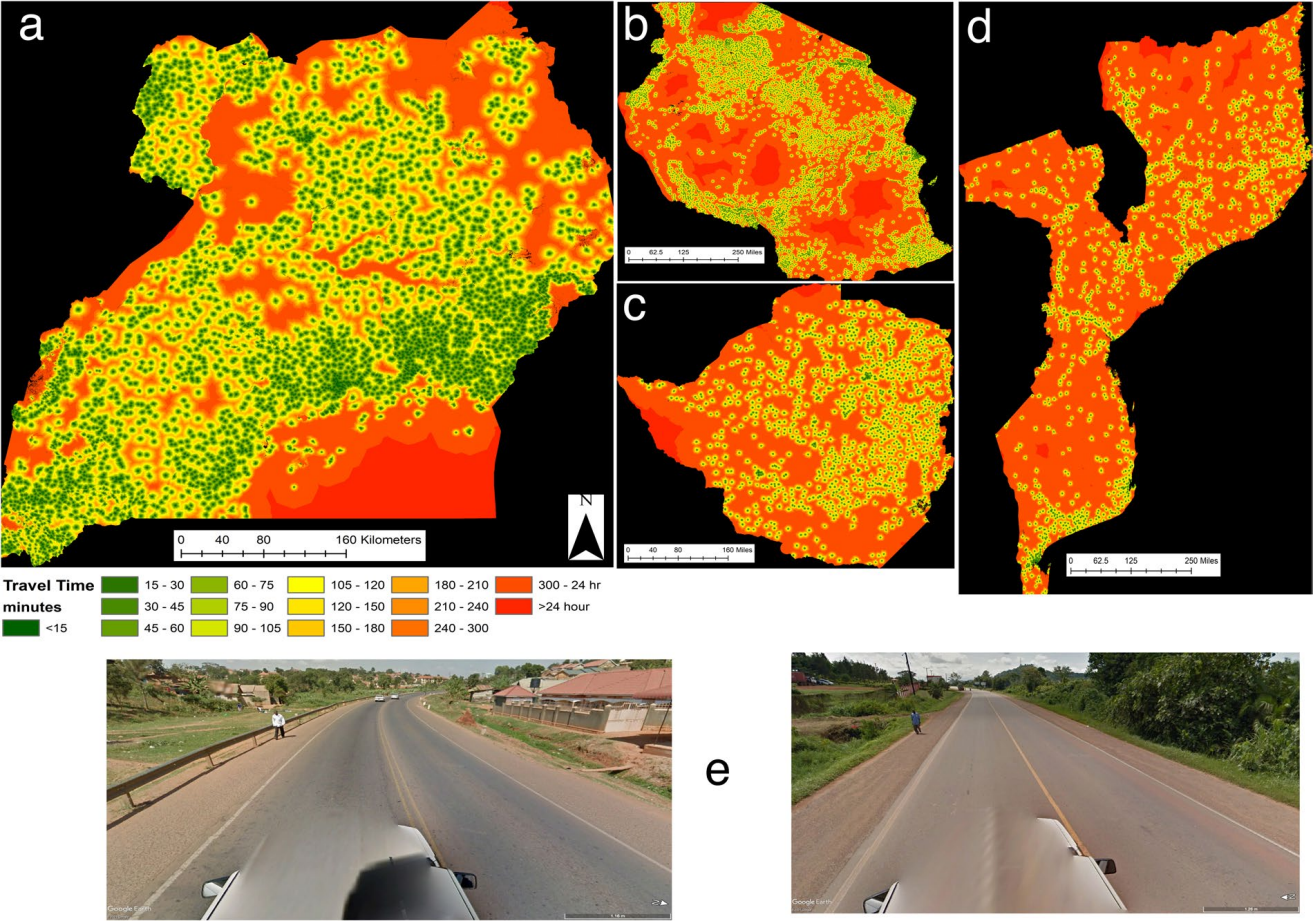
Travel time to any health centre in (**a**) Uganda, (**b**) Tanzania (**c**) Zimbabwe and (**d**) Mozambique calculated using the CPAS method and produced on a 20 m raster grid. These data are available for download (see Supplementary Table B). Google street view images showing pedestrians using the sides of major roads and highways in Uganda (**e**), included to demonstrate that a walking speed of 5 km/hr is appropriate for highways.

Published walking speeds specific to road surface and land cover type for modelling access to services for Niger^23^ and the Horn of Africa^24^ were used as the baseline for speed estimations in Uganda. We used the maximum walking speed of 5 km/hr (1.39 m/s) that is commonly used to be representative of adult walking speeds^19,23,24^. Speeds ranged from 1.0 km/hr to 3.0 km/hr for non-road and path pixels such as grassland, cropland (Table 1) and from 3.5 km/hr to 5.0 km/hr for paths and road (Table 2). Speeds were fastest on main roads such as highways, primary roads and trunk roads. Pedestrians often walk along the side of these main roads (Fig. 2e) in many countries including Uganda, Mozambique and Zimbabwe as they are easier to traverse than unsealed roads, footpaths and other land cover types. Therefore, we preferentially pushed pedestrians to walk along these roads.

**Table 1:** Sentinel-2 land cover categories and associated walking speeds assigned with and without children.

Sentinel-2 Land Cover Categories	CCI Land cover Code	Walking Speed (km/h)	Walking speed weighted for children (22% reduction)
Trees	1	1.50	1.17
Shrubs	2	1.50	1.17
Grassland	3	3.00	2.34
Cropland	4	3.00	2.34
Often Flooded	5	N/A	N/A
Sparse Vegetation	6	3.00	2.34
Bare Areas^1^	7	1.15	0.89
Built-Up	8	1.50	1.17
Open Water	10	1.00	0.78
No Data	200	N/A	N/A

^1^Areas identified as ‘Bare Areas’ within the Sentinel-2 LULC 2016 for Uganda were primarily dry riverbed or sandbanks within rivers.

**Table 2:** Open Street Map (OSM) road categories provided in the data downloads from OSM and Map with AI.

OSM Roads Feature Class	Count (Total)	Notes (Ministry of Works and Transport - The Republic of Uganda, 2012; Ramm, 2019; OpenStreetMap, 2020)	Walking Speed (km/h)
motorway	105	A restricted access major divided highway, normally with 2 or more lanes plus emergency hard shoulder.	5.0
motorway_link	20	Connecting slip road/ramp	5.0
Trunk & Trunk_link	370 + 91	A main road with a motorway-like layout with multiple lanes which is restricted to motorised vehicles. Unlike motorways, trunk roads might have crossings or traffic lights. Their surface is always tarmacked.	5.0
Primary & primary_link	973 + 57	National roads connect the most important cities/towns in a country. These may be tarmacked and show centre markings.	5.0
Secondary & secondary_link	1991 + 47	Major transportation routes connecting cities and large towns. May be tarmacked but often not.	4.5
Tertiary & Tertiary_link	5460 + 62	These are busy through roads that link smaller towns and larger villages. Most often unpaved, but wide enough to allow two cars to pass safely.	4.5
residential	483527	Roads lined with housing in urban or village areas and where roads do not serve a through connection function.	4.0
unclassified	179029	Minor collector roads that link settlements. These roads are usually unpaved and are only wide enough for one vehicle. Primarily in rural areas and outside of inhabited places, though unclassified roads can be used to link suburbs in a city or town.	3.5
track	80217	This tag is usually used for roads providing access to agricultural or forestry facilities. In Africa, roads within National Parks mostly qualify as tracks too.	3.5
track_grade1-5	644	Solid, usually paved or sealed	3.5
service	14557	Driveways, entrances, private roads, service roads for industry etc	3.5
Bridleway & cycleway	38	Visual inspection shows no specific features to identify as bridleway	3.5
living_street	325	Streets where pedestrians have priority	4.0
pedestrian	121	Pedestrian only streets	4.0
path	153656	Paths are usually impassable for motorised vehicles but may be passable by motorcycles.	3.5
footway	8857	Pedestrian only, separated from parallel highway for vehicles. Can often be obstructed by traders and motor vehicles.	3.5
steps	176	Pedestrian only by nature	3.5
unknown	1507	Unknown type	3.5

Associated walking speeds assigned in the CPAS software are listed. These are based on previous speeds assigned in other studies. The speeds can be changed easily in the CPAS software to reflect local variations or the availability of motorised transport.

In the current work we have focused on child walking speeds since our work in Uganda seeks to understand the impact of access to health centres on multidimensional childhood poverty. Therefore, we reduced walking speeds to account for adults accompanied by children. Bouterse and Wall-Scheffler^22^ observed that unloaded adults travelling alone had an average speed of 1.001 m/s which decreased to 0.773 m/s when they had to carry a child or to 0.785 m/s when older children were present that did not require carrying (they were not counted as a load but were accompanying the adult). Thus, the approximate decrease in travel speed was 22% and we reduced the speeds proposed by^23^ by 22% in the child travel time maps. The speed reduction can be easily switched off or adapted in the software^25^.

### Data acquisition

Data sources were selected based upon resolution and coverage with fine spatial resolution data preferred. As it was intended for the research to be applicable over multiple countries in which UNICEF is working as well as being user friendly for the wider scientific community, data sources were selected which had at least continental Africa coverage. The three data sets required for the travel-time maps are described below and in Supplementary Table A.

- Land cover – Land cover was derived from the Sentinel 2 CCI Land Use Land Cover (LULC) 2016 (20 m resolution) data product http://2016africalandcover20m.esrin.esa.int/^26–28^, (Supplementary Table A). Other products with a greater number of land cover classes were available but only for a limited number of countries. The larger number of land cover classes was of little use as walking speeds were only available from the literature for a limited number of land cover types.
- Elevation – Shuttle radar topography mission (SRTM) Digital Elevation Model (DEM) no-void filled data at 30 metres resolution^29^, (Supplementary Table A) is available globally. Finer resolution datasets were available in some places, however, the use of the SRTM 30 m data allows for standardisation of the elevation data globally.
- Roads – OpenStreetMap (OSM) roads data https://www.openstreetmap.org (Supplementary Table A) is available for most countries globally^30^. Open street map is known to have inconsistent coverage due it being created using volunteered geographic information^31^. Thus, it was merged with the OSM mapwith.ai project (https://github.com/facebookmicrosites/Open-Mapping-At-Facebook/wiki/Available-Countries) data which used AI and high-resolution imagery to identify roads missing in OSM data to produce a more complete road dataset. The standard OSM road data and the mapwith.ai road data are available globally.

### Rasterizing roads

The roads data from OpenStreetMap^30^ is crowdsourced geographic data uploaded by volunteers^31^. This means completeness varies spatially both across and within countries, with Uganda being estimated to be 70% complete^31^. The m*apwith.ai* project by Facebook in conjunction with OpenStreetMap, have applied artificial intelligence alongside human validation to add missing roads and footpaths to the OSM dataset. The roads in both OSM and *Mapwith.AI* datasets were merged using ArcMap 10.7.1^32^ ‘merge’ function in the ArcToolBox. The merged data required a new ‘tag’ attribute to be created which contained the road type and is the only editing a user is required to perform prior to running the code on a new location.

There are up to 29 types of road and path categories in the merged road data and walking speeds for each were derived from speeds used in existing studies (Table 2). Roads categorised as ‘motorway’ or ‘trunk’ are restricted to motorized vehicles according to OSM metadata. However, pedestrians were seen on the sides of some of these roads in Google Maps Street View (Fig. 2e). Therefore, walking speeds were allocated for all road types including motorways (Table 2). Roads are generally considered to be faster for travelling, but also most likely to be used by those walking as they allow easier navigation, therefore the walking speeds assigned to roads were faster than the walking speeds assigned to different landcover types.

The roads data were converted from a polyline shapefile (vector) to a raster surface so that they could be combined with the land cover and elevation data. Vector to raster conversion of roads can introduce issues related to the Modified Areal Unit Problem^33^ as linear data is aggregated into the grid format of the raster surface^34^. When using a coarser cell size, it is more likely that multiple roads will be present inside the same cell. For example, when using a 1 km cell size Delamater *et al*.^16^ found that road coverage was overestimated which would result in faster travel time estimations. The overestimated road coverage derives from simplifying the landcover within each 1 km pixel from multiple ground features (different types of road, paths and other landcovers) to a single feature. Often the fastest road type is prioritised when rasterizing multiple polygons into a single grid cell as was performed in Weiss *et al*.^15^ and in our approach. This overestimation of road coverage will result in overestimation of access or underestimation of travel time to services. Further simplifications such as over connections of roads that result from smoothing the complex edges of polygons and reducing the sinuosity of the roads will also contribute to the overestimation of access or underestimation of travel times.

A 20 m cell size was used for the OSM roads in Uganda to match the CCI land cover data. While 20 m is still wider than many roads it is likely that the impact of time taken to reach the road from within a 20 m cell would be lower than larger cell sizes. Overlapping roads were prioritised by speed with the fastest roads given priority but this can be turned off in the software^25^. However, due to the fine resolution cell size of 20 m, the raster surface remained representative of the road network with little over connection between roads (Fig. 1) this merged data set is published through the Edinburgh Data Share portal for Uganda^35^, Mozambique^36^, Zimbabwe^37^ and Tanzania^38^.

The cost surface is a scalar field but roads are directional. Rasterising a road implies that a cell can be crossed equally from left to right as bottom to top. At coarser resolution this may be a problem which could be dealt with by using an anisotropic cost surface. Using a 20 m resolution approach it is likely that anisotropic rasters are not required because the pixels adjacent to a road would have a higher cost and therefore the cost surface would preferentially push people in the correct direction.

### Converting land cover data to travel speeds

The nine land cover categories available in the CCI (Trees; Shrubs; Grassland; Cropland; Often flooded; sparse vegetation; bare area; built-up; open water) were mapped to walking speeds from Blanford *et al*.^23^ (Table 1). Open-water was initially coded as NA to create a barrier to travel as it would result in pixels with values of infinity in the least cost path allocation. A secondary cost surface model was created with a travel speed of 1.0 km per hour for the ‘Open Water’ category (the water speed can be changed in the configuration file of the software). This is because many of the island populations in Uganda are unlikely to be able to access services and, in particular healthcare services, without travelling over water. The reason for doing so is that when we use the cost allocation surfaces to estimate travel time we are able to prevent travel across water unless it is absolutely necessary. We define absolutely necessary as those living on islands where there are no health facilities present on the island and therefore, the inhabitants would have to use water to travel. This prevents travel times being calculated across water bodies where there may be no ferry service and forces the model to use roads. These speeds can be altered in the software easily and so other users can experiment with these.

### Weighting speeds using topography

Methods of estimating the impact of gradient on walking speeds such as Tobler’s Formula or the Naismith-Langmuir rule are often used when modelling access to services^39^. Recent adaptations to these have been developed to account for slopes up to 30% or approximately 17°^39^ and slopes up to 100% approximately 45°^39^. Therefore, to calculate the impact of gradient on walking speed we used Eq. 1 from Irmischer and Clarke^40^.

1 $$Speed\left(m/s\right)=0.11+{e}^{\frac{-{(S+5)}^{2}}{2\times 3{0}^{2}}}$$

where, *S* is the slope in percent.

The impact on walking speeds is calculated by computing the ratio of the walking speed for a slope and a flat surface. Slopes over 100% (over 45° gradient) were considered impassable. An assumption was made that those travelling to healthcare services were making a return trip and this was accounted for by taking a mean of the speed for a positive slope and negative slope of the same slope value^39^. The impact of negative and positive slopes is not equal (Eq. 2).

2 $$Speed\;impact=\frac{0.5\times \left(\left(0.11+{e}^{\frac{-{(+S+5)}^{2}}{2\times 3{0}^{2}}}\right)+\left(0.11+{e}^{\frac{-{(-S+5)}^{2}}{2\times 3{0}^{2}}}\right)\right)}{0.11+{e}^{\frac{-{(0+5)}^{2}}{2\times 3{0}^{2}}}}$$

### Assembling the cost allocation surface

Two cost allocation surfaces are created using the above methods and are included in the data downloads for each country (Supplementary Table B). The first defines open water as a barrier to travel and so the speed allocated to this landcover is ‘NA’. The second defines open water with an associated speed. To create a walking speed array, first the road walking speeds were used and then missing values were filled with landcover walking speed values. This walking speed array was multiplied by the slope impact grid. The speed for each cell was converted from kilometers per hour to meters per second. Finally, the time (in seconds) to walk across each cell was calculated using Eq. 3 where distance is equal to cell size (20 m). These travel time or friction surfaces as they are also known are published and made available through the Edinburgh Data Share Portal^41–44^.

3 $${\rm{Travel}}\;{\rm{time}}\;{\rm{over}}\;{\rm{cell}}={\rm{cell}}\;{\rm{size}}/({\rm{speed}}\,\ast \,{\rm{speed}}\;{\rm{impact}}\,\ast \,(1-{\rm{child}}\;{\rm{impact}}))$$

### Least cost path analysis for estimating travel time to health facilities

A World Health Organization (WHO) validated spatial database of health facilities in sub-Saharan Africa^45^ was used to indicate the location of health services in Uganda. The data were clipped to Uganda using GADM administrative boundary level-0 data. Health facilities with invalid locations such as those outside Uganda or located on water are identified and removed in the software^25^ and a report is generated in csv format. Healthcare facilities which were located within cost surface cells that had been deemed impassable were identified and if adjacent cells were passable were randomly moved to one of these or if no adjacent cells were passable then the location was removed from the analysis, these facilities are also identified and included in a report produced by the software^25^.

The cost allocation surface and health facilities locations are combined using the least cost path method to calculate travel time from each cell in the array to the nearest health facility. The code first uses the cost allocation surface with water having an NA for travel time. Where travel time to healthcare services was measured as infinite, this indicated cells that had to traverse water in order to reach a health facility. In these cases, the travel time was measured using the water passable cost allocation surface in our example this meant the water had a travel speed of 1 km/hr applied (Table 1).

### Type of health facilities

**Fig. 3:**
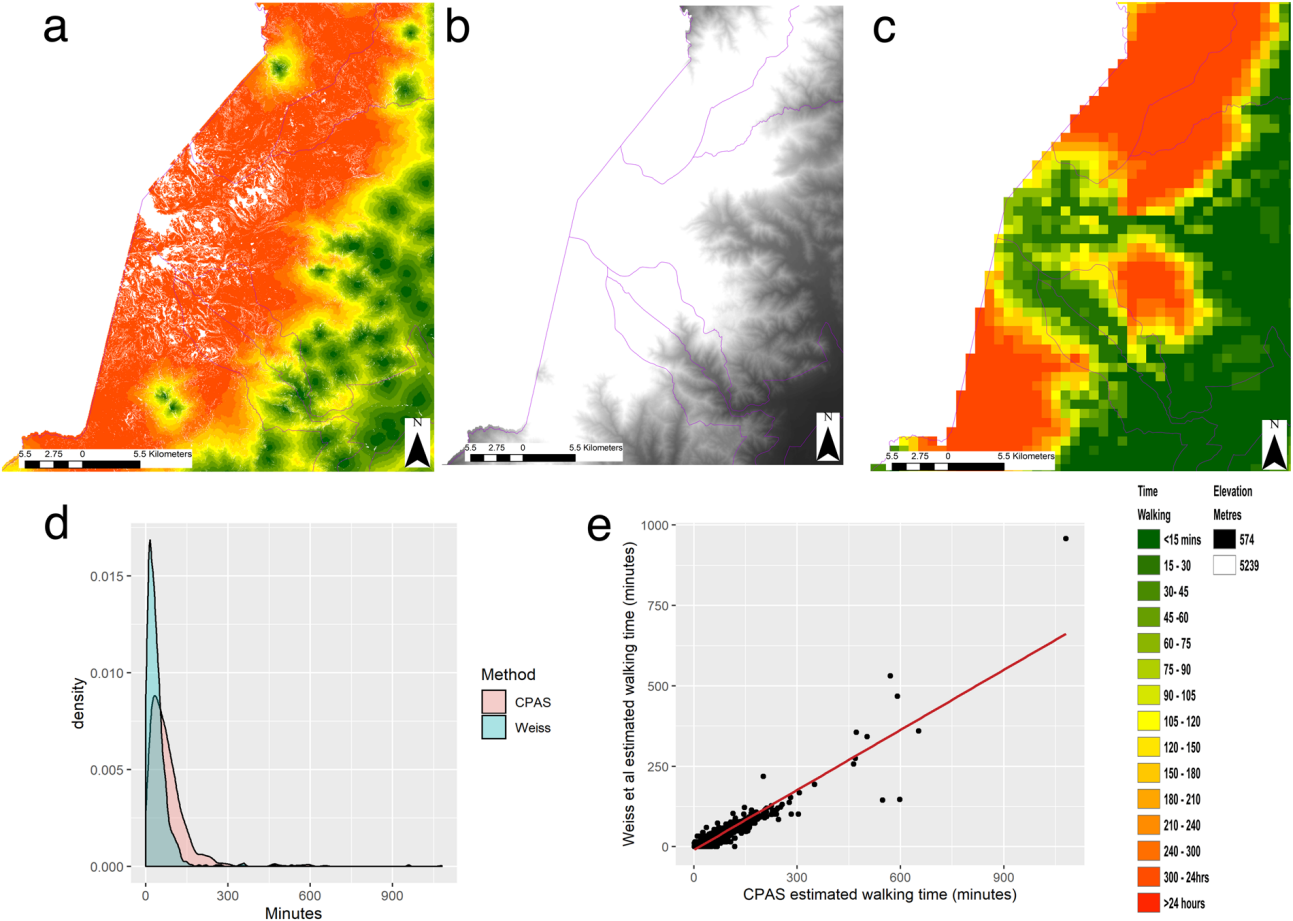
(**a**) Travel time to any clinic in minutes calculated using the CPAS 20 m method. (**b**) Travel time to clinics and hospitals using the method from Weiss *et al*.^16^ and a 1 km grid (**c**) Elevation data derived from the Shuttle radar topography mission (SRTM) showing mount Stanley. (**d**) Density plots showing the distribution of travel times for 682 Demographic and Health Survey Clusters across Uganda for both CPAS and Weiss *et al*. methods. (**e**) Scatterplot showing the monotonic relationship between the travel times estimated by the CPAS and Weiss *et al*. methods. Note in 3E the point at the top-left is not an outlier, it is a DHS cluster located on an Island with no health centre recorded in the WHO data. The white pixels in (**a**) indicate no data and result from CPAS removing any pixels with a slope >45 degrees. Using a 1 km grid as in (**c**) results in aggregation of slope angles and therefore there are unlikely to be any pixels with slipes >45 degrees. This results in the 1 km grid approach producing faster travel time estimates in the steepest cells which in reality are likely to be avoided by pedestrians^65–69^.

We provide several travel time maps for each country one of which is the travel time to the closest facility of any type (Figs. 2a, 3). Since this is a simplification of the reality of accessing health facilities, we split the health facility data by type and calculate travel times to each. For each country there are different facility types in the Maina *et al*.^45^ data thus we describe below briefly which facility types have been used in Uganda, Tanzania, Zimbabwe and Mozambique.

The health facility types available in the Uganda data were: clinics, health centres (Level II, III and IV), hospitals and regional referral hospital. We used the Uganda Hospital and Health Centre Survey Census^46^ to identify the services available at each facility type. Level II centres were excluded as they provide only basic medical care, whereas level III and IV facilities provide maternity care, basic lab services and inpatient care. Level IV facilities are the only facility type to have qualified doctors^46^. Hospitals represent access to surgical care and specialist health services. Therefore, in Uganda we have produced the following maps of travel time to: (1) any type of facility^47^ (Fig. 2a); (2) level III health centre^48^; (3) level IV health centres^49^, and; (4) level IV health centres and hospitals^50^. Regional and national referral hospitals were excluded as to access these facilities a patient must be referred from another health facility (Level IV). Clinics were excluded because they are undefined in the census document, although we did not calculate travel time to Level II facilities these are the most frequent and therefore are covered in the map of travel time to any facility.

The health facility types available in the Zimbabwe data were; clinics, rural health clinics, rural hospital, district hospital, provincial hospital and central hospital. We used the USAID supported Zimbabwe Health System Assessment^51^ to identify what type of services were available at each facility type. Rural health clinics and hospitals provide basic preventative medicine, maternity care and curative services. District hospitals provide primary health care services and can treat emergencies. Therefore, in Zimbabwe we have produced the following travel time maps to: (1) any health facility (Fig. 2c)^52^ which includes all the health facilities available in Zimbabwe from the Maina *et al*. (2019) data; (2) rural health centres^53^; and (3) rural and district Hospitals^54^. Clinics were excluded from some of the maps as, despite several clinic types being mentioned in the literature, it was not possible to differentiate these types in the data. However, since they were the most common facility type in Zimbabwe they are included in the maps to any facility type.

The health facility types in Tanzania were; dispensary, health centre, hospital, district hospital, designated district hospital, referral hospital and national hospital. We used the Health Sector Strategic Plan July 2015 – Jun 2020^55^ to identify the type of services available at each facility type. Dispensaries provide the most basic of services and do not provide inpatient care. Health centres can admit patients and sometimes provide surgical services. Hospitals and district hospitals provide medical and basic surgical services. Referral, regional referral and national hospitals provide specialist medical care and medical training. Therefore, for Tanzania we have provided the following travel time maps to the following types of health facility: (1) dispensary (Fig. 2b)^56^; (2) health centres^57^ and (3) hospitals and district hospitals^58^.

For Mozambique we provide a single travel time map to any health facility (Fig. 2d) listed in the Maina *et al*.^45^ database^59^. At the current time it was not possible to identify the services offered at each type of clinic but these will be updated in the near future.

## Data Processing Software

- Python 3.
- Python - required packages: fuzzywuzzy; numpy; pandas; osgeo; geopandas; skimage; xarray; rioxarray; Fiona; rasterio – these get installed automatically using the setup script or can be installed into a conda environment using the environment file.
- ArcMap 10.7.1

## Data Records

All data generated in this study are available at Edinburgh Data Share^35–38,41–44,47–50,52–54,56–59^. Supplementary Table B provides the descriptions of the data shared on Edinburgh Data Share and DOIs for each of the datasets as part of the submission. These data include the children’s travel time to any health centre, the children’s travel time to specific health centre types (3 additional maps in Uganda and 2 additional maps in Tanzania and Zimbabwe). We also provide the cost allocation/effort surfaces for children and the health facility locations (Supplementary Table B). The external data sources used for generating the cost surface are listed in Supplementary Table A.

## Technical Validation

To validate the travel times from the CPAS software we have compared the travel times from CPAS with those estimated by Weiss *et al*.^14^ at 1 km spatial resolution which have been validated against GoogleMaps API data in the past^60^. We used 682 village clusters in the Ugandan 2016 Demographic and Health Survey (DHS)^3^. We overlaid village clusters onto both cost surfaces and used the ‘*extract to point*’ function in ArcMap 10.7.1^32^. We created summary statistics, density plots and ran a Spearman’s rank correlation to compare the travel times estimated by the two approaches. A scatterplot (Fig. 3e) shows that the two travel time estimations are monotonically related which is further supported by the Spearman’s rank correlation between the two walking speeds (rho 0.89 and p < 0.01). The density plots (Fig. 3d) indicate that the CPAS method estimates a wider distribution of travel times than the Weiss *et al*. method. The walking times estimated in both the Weiss *et al*.^14^ 1 km and the CPAS 20 m approaches are skewed towards lower values (Fig. 3d) which is expected as there will be more health centres located near to populated areas. Those clusters with higher estimated travel times are either located on Islands in Lake Victoria or in isolated mountain regions of Uganda.

Comparing the distribution of travel times and correlation indicates that there is strong agreement between the two methods as to which clusters have the greatest access to health facilities. The differences lie in the absolute values or the total minutes estimated for walking to the nearest health centre. Summary statistics show that the CPAS walking times have no zero estimated walking times (Table 3). This is because at 20 m resolution it is highly unlikely that a village/cluster will fall within the same grid cell as a health centre. In comparison, the Weiss *et al*.^14^ estimations have 72 clusters estimated to have zero minutes travel time (Table 3). This is due to the aggregation of the landscape using a 1 km resolution grid and the smoothing involved in this approach, as zero minutes in Weiss *et al*.^14^ could translate into a travel distance of 0–1000 m whereas in the CPAS approach a 0-minute travel time would only occur if the village cluster was 20 m or less from the health centre.

**Table 3:** Summary statistics for the walking only travel times from the 682 clusters to the nearest health centre calculated using the 1 km Weiss *et al*.^16^ and 20 m CPAS methods.

	Min	1^st^ quartile	Median	Mean	3^rd^ quartile	Max
1 km Weiss *et al*.^16^	0	15	30	40.85	50.50	958
20 m CPAS	1	33.03	62.76	81.55	102.15	1080

Calculated using 682 clusters from the 2016 Ugandan Demographic and Health Survey (DHS).

The median and mean estimated travel times (Table 4) are roughly twice as large in the CPAS travel times than the Weiss *et al*. travel times (Table 3). This further support conclusions from Delamater *et al*.^16^ that a larger grid overestimates road coverage and therefore underestimates travel times. This is due to the simplification of road sinuosity in larger grids that is occurring in the Weiss *et al*.^14^ 1 km estimations allowing roads and particularly the fastest roads to be overrepresented over-connected compared with other land cover types (Fig. 1b,c).

**Table 4:** The number of village clusters that were within given walking times of the nearest health centres for the 1 km Weiss *et al*.^15^ and the 20 m CPAS method.

	Weiss *et al*.^16^ (1 km)	CPAS (20 m)
0 minutes	72	0
< = 15 (including 0)	227	50
>0 & < = 15	155	50
< = 30	344	159
>0 & < = 30	189	109

Calculated using 682 clusters from the 2016 Ugandan Demographic and Health Survey (DHS).

The overall differences between the two approaches are considerable when considering how many village clusters are reported as being within 30 minutes of a health centre. According to the Weiss *et al*.^14^ method roughly half (344/682) of DHS clusters are within a 30-minute walk of the nearest health centre. But if using the CPAS method this drops to a quarter of DHS clusters (159/682). It is expected that, given the improvement in sinuosity of roads (Fig. 1b), the addition of roads and footpaths previously missed in OSM data and the finer resolution (Fig. 3), the CPAS method is more accurately characterising travel time by pedestrians. This could have large implications for public health planning in Uganda. If using the finer resolution approach in CPAS and targeting a 30-minute travel time then potentially the 1 km grids produced by Weiss *et al*.^14^ are overestimating access or underestimating the time it takes for people to walk to a health centre. Further work is needed to fully validate these travel speeds using ground-based surveys which were not possible during this study due to travel restrictions imposed by the COVID-19 pandemic.

The validation detailed above did not make use of ‘ground-truth’ data. We examined the Uganda DHS^3^ data as it sometimes contains information on travel times to health facilities. However, the DHS data for Uganda in 2016 did not contain this information. Conducting our own survey was not possible due to budget restrictions and the COVID-19 pandemic preventing UNICEFs ground team performing validations. Further, there is uncertainty in which health centre households are travelling to. Our model as well as almost all other similar approaches assume travel to the nearest facility. But we know from previous field work in other countries that households choose to go to different health facilities for a variety of reasons and often these are not the nearest facilities to the village but along a route that the individual takes when carrying out other activities for example. Thus, there is limited opportunity for validation at this time.

All of the input data that we have used has been independently validated. The walking speeds we have used have been published in peer-reviewed journal articles. Each of the component data sets have been validated or examined for completeness by others in the literature.

- The ESA CCI 2016 Land Cover data for Africa is classed as a prototype dataset and has been independently validated^61^. The validation found that the land cover accuracy was highly variable across Africa with an average overall accuracy of 65%. In Uganda the accuracy ranged between 46% and 65% due to misclassification errors between grassland and cropland. This overall accuracy is low, however, the highly detailed roads data available from OSM and mapwith.ai meant that the land cover map was less important for overall estimates of travel speeds. Furthermore, the land cover can be improved in the future or can be replaced in the code with a user defined Sentinel-2 land cover classification.
- The OSM data is continually validated during the digitisation process by OSM. Road completeness varies spatially both across and within countries, with Uganda being estimated to be 70% complete in 2017^31^. However, this completeness was increased by merging the roads with the mapwith.ai project footpaths and roads.
- The mapwith.ai data was manually validated prior to release. However, there were no published estimates of the completeness of roads and footpaths across Uganda at the time of this writing.
- The health facility location data were validated before being published^45^.

## Usage Notes

Currently, all the input datasets use geographic coordinates which get converted into metres using a constant factor so that a cartesian coordinate system can be used to compute the gradient. The code assumes a uniform factor to convert degrees latitude and degrees longitude into metres. This approximation works near the equator but may be problematic further away from the equator. However, the gradient is used to modify the cost which is an approximate model so it will have a small impact on the final cost allocation and travel time estimations. The code is open and licensed in such a way that users could adapt it to accept different projected coordinate systems should they see fit.

The cost allocation surfaces provided can be used in GIS analysis to estimate time to travel to user selected services. The user would need some form of location information for a particular service such as health centre, market, school, city or all-weather road for example. Thus, these surfaces can be used by others for further analysis when considering access to services across different countries.

No editing is required of the ESA CCI land cover data prior to running the code, it just needs to be downloaded and clipped to the country boundaries. Users need to ensure that the landcovercosts.csv file has the correct land cover class codes and required speeds specified. These land cover classes are the same as those in the ESA CCI land cover product so if a user opts to use a different land cover dataset the land cover class values will need to be changed.

The 20 m spatial resolution used in this analysis does mean that some files are very large, preventing the software being converted into an online web platform currently. High-performance computing is required for larger countries such as Tanzania and Mozambique. If users have a low bandwidth and cannot run the software they can make reasonable requests to the corresponding author to calculate travel times to particular facility locations of their choosing. The files are large due to being 20 m spatial resolution:

- Uganda– 6.7 gb
- Tanzania - 26 gb
- Zimbabwe – 11.6gb
- Mozambique – 37.9 gb

### System requirements

The code works out of the box on an Ubuntu 20.04 system. For analysis using data for Uganda the code required 50GB of memory. For analysis in larger countries the code required 128GB and took approximately 3 hours to run for Zimbabwe, 4.5 hours for Tanzania and 6 hours for Mozambique.

The CPAS software works out of the box in that only standard Python packages and dependencies are required for it to run. These packages can be installed into either a conda or standard python virtual environment. We have run the software on Linux however, it is not restricted to this OS and we have tested in LTS ubuntu, but it will work on windows/mac/other linux with a suitable python installation. ArcMap was used for some of the preprocessing, but this could be done done in QGIS or GRASS or R if a user does not have access to ESRC ArcMap Licenses. It is possible to distribute the CPAS software as a docker container with a user-friendly graphical frontend similar to AccessMod5. However, due to of the high-resolution of the datasets, significant computational resources are required to compute them.

## Supplementary information

Supplementary Table A

Supplementary Table B

## Data Availability

The software/code works out of the box on a current Ubuntu system using Python, and all source data were derived from open and free datasets available for the whole of Sub-Saharan Africa. The Python software^25^, scripts and a virtual environment are provided with the data and requires minimal input from the user. The software is controlled by a configuration file. The GitHub repository contains an example configuration file, creation.cfg. In this file the following should be changed: • Path to input data sets • Names of input data sets should these have been changed. • Path to the location where the output files should be saved • Names of the output files. The user can also choose to change the following settings in the configuration file: • Walking speed reduction to account for children. As standard it is set to 0.78 to generate a 22% reduction in the travel speeds. If a user wishes to generate a cost allocation surface for adult walking speeds they should change the factor to 1.0. • Water speed can be changed in this file. It is set to NA in the landcover.csv and should remain as such. The water speed can also be changed in the configuration file and will appear in the water passable cost allocation surface. Additional steps required prior to running the code: • The roads from the standard OSM roads download and m*apwith.ai* download should be merged. We used the merge function in ArcMap 10.7.1^32^. The merged road data require a new ‘tag’ attribute to be created which contains the road type (name). • The user should check the roadcosts.csv file to ensure the road names match those in the shapefile. • The user can change the travel speeds assigned to each road type by editing the roadcosts.csv file. • The user can change the travel speeds assigned to each land cover type by editing the landcovercosts.csv file. If users want to use a different land cover map they can do so by ensuring that the land cover types in the landcovercosts.csv match those in the chosen land cover data. All of the analysis was conducted using Python-3 apart from merging the OSM roads with the MapwithAi roads. The code has been developed as an all-in-one software which can be downloaded from Zenodo^25^ and GitHub [https://github.com/ChildPovetyAccesstoServices/cpas/tree/v1.0] and is licensed under the GNU General Public License v3.0 only, meaning changes to the code are permitted as long as they are distributed under the same license. The code includes an example configuration file **creation.cfg***.* This is the only file the user needs to alter to repeat the process. Within this file the user specifies the location of the input data and the names and paths for the output files. The user can also specify the weighting if they wish to consider children’s travel speed, if not they can set the reduction factor to 0 which will generate adult cost allocation surfaces. The code runs in two steps (1) calculate the cost allocation surfaces (2) use the least cost path analysis to estimate the travel time from every pixel to the nearest health centre. All rasters are read and written using the rasterio python package (https://github.com/mapbox/rasterio) together with xarray^62^. The steps and python packages used for pre-processing the roads data: • Walking speed values are assigned to an array based on land cover type. • A CSV file of OSM road categories and associated walking speeds was used to input the assigned walking speeds into a pandas Data Frame^63^. • A shapefile containing roads was rasterised using the walking speeds assigned to each type of road. The fastest walking speeds are used for pixels that contain roads of different types. There were a few steps to this which are detailed below: a. The road types of the CSV file were matched to the road types of the shapefile using a fuzzy match to ensure a walking speed was assigned to every road type in the shapefile. b. The road types were grouped by walking speed. Each group of roads was processed separately. c. Roads from the shapefile were filtered by road type and rasterised using the rasterize function of the rasterio.features module. The walking speed is used as value for the rasterised roads. The all_touched option was used so that all grid squares in which road segments existed were counted as road segments in the new raster. This was essential so the rasterization of the roads retained the connections. d. The resulting walking speed surfaces were merged taking the maximum value at each pixel for all road types. Pre-processing steps of the SRTM data required: • Re-sampled to 20 m resolution, using bilinear interpolationSlope is calculated using the formula$slope=\sqrt{\left({\left(\frac{dH}{dx}\right)}^{2}+{\left(\frac{dH}{dy}\right)}^{2}\ast \frac{100}{111120}\right)}$ The factor 100 converts the gradient to a percentage slope and the factor 111120 coverts degree latitude/longitude to metres. This approximation is only valid close to the equator which is the case in this study. As Eq. 1 does not account for gradients over 100%, these were removed and replaced with a NA value. The cost allocation surface and health facilities locations were combined to calculate travel time from each cell in the array to the nearest health facility using the graph.MCP_Geometric() and find_costs() methods from the SciPy package^64^.
